# A successful twin healthy live birth achieved from eggs with a rare perivitelline space abnormality: A case report

**DOI:** 10.18502/ijrm.v21i3.13202

**Published:** 2023-04-14

**Authors:** Daniele Ferri, Domenico Baldini, Giorgio Maria Baldini

**Affiliations:** Momo Fertilife Clinic, Via Cala Dell'Arciprete 2C, Bisceglie, Italy.

**Keywords:** Oocyte, Granulosa cell, Intracytoplasmic sperm injection, Perivitelline space, Case report.

## Abstract

**Background:**

Phenotypic dysmorphism is not rare to be found in the human oocyte, especially in the perivitelline space, which are among the most important aberration of the extra cytoplasmic component.

**Case Presentation:**

The case is of a 30-yr-old woman with no previous pregnancy, attempting an in vitro fertilization treatment for the first time. Given the extraordinary quantity of granular particles found in the perivitelline space, visible after the stripping procedure, it was not possible to establish the presence and position of the first polar body to appreciate the correct oocyte maturation (metaphase 2). Nevertheless, all the eggs were injected by the intracytoplasmic sperm injection. A time lapse incubator was used to perform the entire culture. Hence, a record of 6 days culture video was obtained. Only 2 eggs could fertilize correctly and reach the blastocyst stage on day 6. The embryos were frozen and subsequently transferred as frozen embryo transfer following the next menstrual cycle.

**Conclusion:**

The exceptional presence of granular particles in the perivitelline space, which reminds us for aspects and behavior of the granulosa cells, seems to affect the fertilization but not the blastocysts quality. As a matter of fact, the woman, after the embryo transfer, achieved a successful twin live birth.

## 1. Introduction

Between 60-70% of the retrieved oocytes after pick-up has reported to exhibit one or more phenotypic dysmorphisms, normally classified as extra cytoplasmic or cytoplasmic (1, 2).

Perivitelline space abnormalities are among the most important dysmorphism of the extra cytoplasmic component, but the fate of these anomalies remains unclear, giving contradictory results in the literature (2).

Here we report a successful case of a woman having a rare perivitelline space anomaly consistent in inclusion of what we believe to be granulosa cells, exhibited in most of the eggs, which, after intracytoplasmic sperm injection and frozen embryo transfer, the woman achieved a twin pregnancy and live births.

## 2. Case Presentation

A 30-yr-old woman with no previous pregnancy, attempting for the first-time treatment of in vitro fertilization, came to our clinic in November 2019. A testicular germinoma was previously found in the husband which caused the removal of the left testicle. A reason of infertility for this couple was caused by the low motility of his sperm. Using recombinant-follicle stimulating hormone (Gonal-F ${}^{\text{TM}}$ ; Merk), the ovarian stimulation was performed beginning from the second day of the menstruation cycle. Since the woman was a hyper responder (anti-mullerian hormone: 8.46 ng/ml), the gonadotropin releasing hormone antagonist (Cetrotide ${}^{\text{TM}}$ ; Serono) was used to induce a trigger. To induce ovulation, a trigger for gonadotropin releasing hormone agonist (Suprefact ${}^{\text{TM}}$ ; Sanofi) was given when most of the size of the follicles reached 18 mm. Between 35 and 36 hr later an ultrasound-guided oocyte pick-up was performed. 15 oocytes were retrieved, and the stripping procedure was performed about 2 hr after the egg collection.

Different grades of inclusions in the perivitelline space (PVS) were found in 13 eggs out of 15. Given the exceptional quantity of inclusions in the PVS of most of the oocytes, it was not always possible to establish the presence and position of the first polar body to appreciate the correct oocyte maturation (metaphase II); however, the intracytoplasmic sperm injection was performed for all the eggs around 30 min after the denuding procedure. Only one egg in the entire cohort showed no visible abnormality. The entire embryo culture using a one-step medium (G-TL ${}^{\text{TM}}$ ; Vitrolife), was performed using a time-lapse incubator (Geri ${}^{\text{TM}}$ , Genea). Therefore, a full video of 6 days of culture was recorded. After 12 hr of culture we could observe a distinctive behavior in the PVS, the pellucide zone was completely broken by the expansion of the granular particles, leaving the subsequent embryo division without it. On day 1, the inclusion spreading allowed the operator to evaluate the eggs fertilization, observing the presence of pronucleus. 2 eggs out of 15 were fertilized correctly, with 2 pronucleus around the center of the cytoplasm. One egg was found to be degenerated, and the rest had no visible pronucleus in the cytoplasm. All the embryos were classified using the Gardner embryo grading system (3).

**Figure 1:**
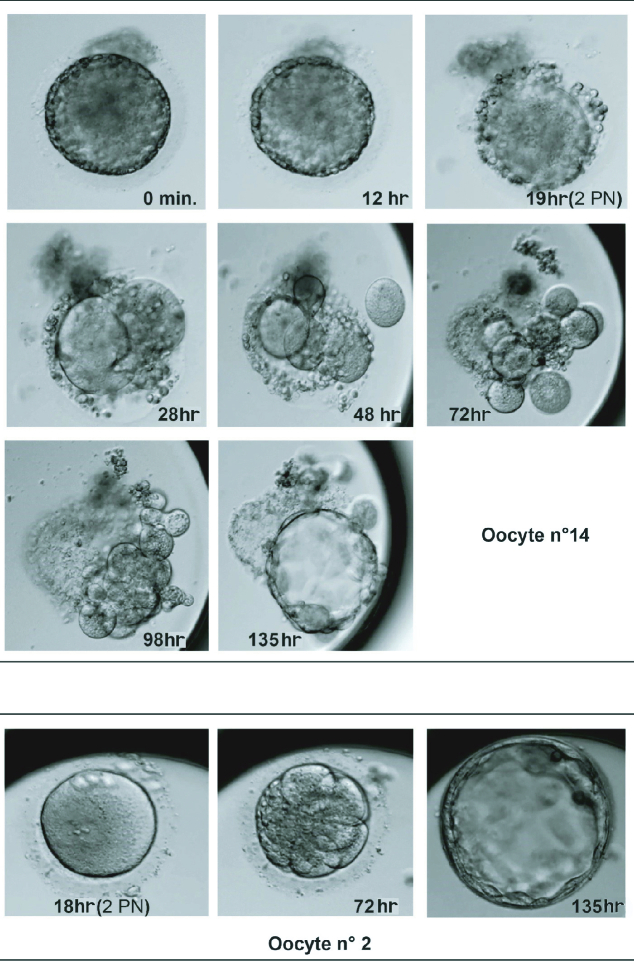
Time-lapse pictures captured from the Geri ${}^{\text{TM}}$ incubator showing some crucial moments of the development of the 2 embryos transferred and implanted.

On day 3 (after 72 hr of culture), we could observe one embryo with 6 cells grade B, and one embryo with 7 cells grade C on day 6 (after 135 hr of embryo culture), the 6 cells grade B reached the blastocyst stage, and it was classified as 5 BB (Figure 1 oocyte n 14), while the second embryo 7 cells grade C reached the blastocyst stage as well, with a score of 5 CC (Figure 1 oocyte n 2). The blastocysts were subsequently vitrified on the same day using a Kitazato ${}^{\text{bad hbox}}$ kit (Kitazato Corporation, Fuji, Shizuoka, Japan). Following her next menstruation cycle, the blastocysts were transferred as a frozen embryo transfer and progesterone was used to support the luteal phase. A pregnancy test 12 days after transfer showed a beta-human chorionic gonadotropin level of 1004 mIU/ml. At the 7 ${}^{\text{th}}$ gestational week, a twin pregnancy with a visible heartbeat and 2 different yolk sacs were detected. 2 baby girls were born weighing 2.6 kg and 2.5 kg at 40 wk of gestation, showing a normal karyotype.

### Ethical considerations

The Local Ethics Committee of the MOMO' FERTILIFE Center has evaluated the present manuscriptand and deemed it ethically justifiable. The committee has recommended that certain conditions be documented and guaranteed, including that the couple participating in the study has given informed consent and that the consent was obtained from an adult who was aware and not subjected to any form of coercion. The committee discussed and approved the ethical aspects of the research project in their session on January 18 ${}^{\text{th}}$ , 2021.

## 3. Discussion 

The oocyte and granulosa cells, secrets 4 glycoproteins (zona pellucida [ZP]1, ZP2, ZP3 and ZP4), which is known to form the pellucide zone during the folliculogenesis in human oocytes (4). In this specific case, we present a cohort of eggs where most of them have appeared, what we believe, to be granulosa cells trapped in the PVS, sometimes covering the egg completely and not allowing an easy assessment of oocyte quality and especially the presence and position of the polar body (which is particularly important to perform an intracytoplasmic sperm injection without damaging the meiotic spindle). Besides, due to the mucification reaction, the cumulus cells secrete a viscous extracellular matrix that dissociates them from one another (5). Furthermore, we noticed that the pressure generated by this phenomenon increases the PVS gradually during the early hours after the injection, stretching the ZP until it breaks, leaving the embryo to develop outside of the ZP. On this link (https://www.youtube.com/watch?v=vN-aPCFve8w), a full video is available, recorded through Geri ${}^{\text{TM}}$ time-lapse, showing the embryo developing of the egg n 14 (Figure 1) after intracytoplasmic sperm injection.

It is difficult to explain the cause of this abnormality and to date, this particular behavior is not yet described in literature.

To the best of our knowledge, only one study conducted with mice knocked out for the *ZP1* gene has shown an engulfment of cumulus cells in the PVS, and those trapped cells had undergone mucification. Subsequently, a granular matrix was observed surrounding them and infiltrating the zona matrix, the absence of the ZP1 protein has resulted in a thinner and more fragile ZP, which can lead to ectopic accumulation of granulosa cells within the PVS (2)*.*

**Figure 2:**
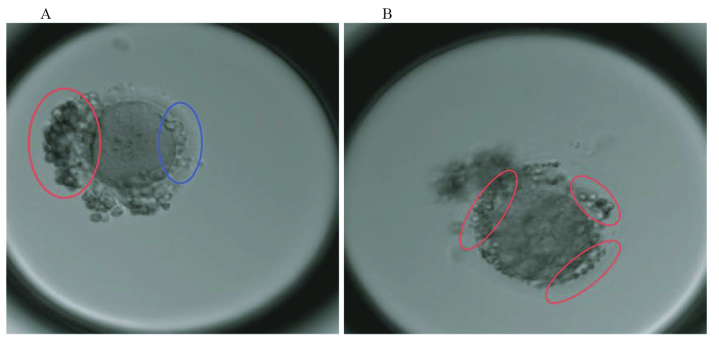
A) A picture taken from the Geri ${}^{\text{TM}}$ Time Lapse incubator shows granulosa cells attached to the ZP (red circle) and in the PVS (blue circle). B) Egg 14 with visible granulosa cells (red circles) in the PVS and 2 PN around the center of cytoplasm. (Pictures taken at 20x magnification).

The time-lapse technology has allowed us to record the complete video of the embryonic development of those eggs and show us a similar behavior described in the murine oocytes experiment, which is still very unique in humans. Moreover, it allows us to differentiate it from coarse granulation or fragment accumulation in the PVS, which has a very different aspect in terms of size, color, and shape (Figure 2A and 2B shows granulosa cells line trapped in the PVS). Besides, ordinary fragments do not lead to any PVS expansion and it does not constantly move during the embryo development.

Since the lack of studies about these particular inclusions, no further comparison can be done.

However, as observed from the video, the absence of ZP did not compromise the development of the embryo, reaching the blastocyst stage at 135 hr from the injection.

In conclusion, this case report wanted to show the extraordinary case of this woman who successfully reached a healthy live birth of 2 baby girls despite the very distinctive oocyte anomaly in most of her eggs, which to date is still not described in the literature of human oocytes.

##  Conflict of Interest

All authors declare no competing financial interest or personal relationships are potentially responsible to influence the work reported in this paper.
